# Genome survey sequencing of *Atractylodes lancea* and identification of its SSR markers

**DOI:** 10.1042/BSR20202709

**Published:** 2020-10-28

**Authors:** Tingyu Shan, Junxian Wu, Daqing Yu, Jin Xie, Qingying Fang, Liangping Zha, Huasheng Peng

**Affiliations:** 1 College of Pharmacy, Anhui University of Chinese Medicine, Hefei 230012, China; 2 College of Pharmacy, Anhui Medical University, Hefei 230032, China; 3 Institute of Conservation and Development of Traditional Chinese Medicine Resources, Anhui Academy of Chinese Medicine, Hefei 230012, China; 4 Research Unit of DAO-DI Herbs, Chinese Academy of Medical Sciences, Beijing 100700, China

**Keywords:** Atractylodes lancea, Functional annotation, Genome size, Genome survey, Simple sequence repeat (SSR)

## Abstract

*Atractylodes lancea* (Thunb.) DC. is a traditional Chinese medicine rich in sesquiterpenes that has been widely used in China and Japan for the treatment of viral infections. Despite its important pharmacological value, genomic information regarding *A. lancea* is currently unavailable. In the present study, the whole genome sequence of *A. lancea* was obtained using an Illumina sequencing platform. The results revealed an estimated genome size for *A. lancea* of 4,159.24 Mb, with 2.28% heterozygosity, and a repeat rate of 89.2%, all of which indicate a highly heterozygous genome. Based on the genomic data of *A. lancea*, 27,582 simple sequence repeat (SSR) markers were identified. The differences in representation among nucleotide repeat types were large, e.g., the mononucleotide repeat type was the most abundant (54.74%) while the pentanucleotide repeats were the least abundant (0.10%), and sequence motifs GA/TC (31.17%) and TTC/GAA (7.23%) were the most abundant among the dinucleotide and trinucleotide repeat motifs, respectively. A total of 93,434 genes matched known genes in common databases including 48,493 genes in the Gene Ontology (GO) database and 34,929 genes in the Kyoto Encyclopedia of Genes and Genomes (KEGG) database. This is the first report to sequence and characterize the whole genome of *A. lancea* and will provide a theoretical basis and reference for further genome-wide deep sequencing and SSR molecular marker development of *A. lancea*.

## Introduction

*Atractylodes lancea* (Thunb.) DC., which belongs to the Asteraceae (Compositae) plant family, has been widely used as traditional medicine in many countries, including China and Japan. The main pharmacologically active components of *A. lancea* are present in the volatile oils and include atractylon, atractylodin, hinesol and β-eudesmol [1,2]. Previous pharmacological studies have demonstrated that the extract and chemical constituents of *A. lancea* have promising anti-inflammatory, anti-cancer, and anti-microbial effects and have been used as a remedy for gastritis and gastric ulcers [3–5]. *A. lancea* also has anti-influenza properties, as atractylon has been shown to effectively kill influenza A virus subtypes H3N2 and H5N1, and influenza B [6]. In addition, *A. lancea* also has immunomodulatory activity [7].

Despite its important medicinal value, the genetic information of *A. lancea* has remained largely unknown. The chromosome number and karyotype of *A. lancea* have been studied. The chromosome number of *A. lancea* is 2*n* = 24, and the karyotype is 2B [8–10]. In addition, the cloning and expression analysis of *DXS* [11], *DXR* [12], *HMGR* [13] and *FPPS* [14] genes in *A. lancea* have been reported; however, the genome size and genome-wide sequencing of *A. lancea* have not been reported. The genomes of Compositae plants, such as *Carthamus tinctorius* [15] and *Artemisia annua* [16], have been sequenced, and these genome studies can provide a reference for the development of these medicinal plants.

Next-generation sequencing (NGS) technology is a relatively new methodology that can enable the identification of large numbers of simple sequence repeat (SSR) markers. This technology has dramatically increased the sequence output while also reducing time and cost [17,18]. Genomic survey analysis is a method of combining NGS technology with K-mer analysis to obtain species genome size, GC content, heterozygosity rate, and repetition rate. This method has been used to accurately predict the whole genome sizes of *Xanthoceras sorbifolium* [19], *Pennisetum purpureum* [20], *Pistacia vera* [21], and other plant species. In the present study, we used genomic survey analysis to study the genome of *A. lancea*. The aims of the present study were threefold; first, to estimate the genome size, GC content, and heterozygosity rates for *A. lancea*; second, to characterize the genome-wide SSRs in the *A. lancea* genome using a genome survey, and third, to perform Gene Ontology (GO), Kyoto Encyclopedia of Genes and Genomes (KEGG), and KOG pathway analyses to further elucidate the main biological functions of the genome survey.

## Materials and methods

### Plant material

Fresh *A. lancea* (Thunb.) DC. plants were collected in July 2018 from Nanjing city, in Jiangsu Province, China (Supplementary Figure S1). Fresh leaves at the apex of *A. lancea* plants were transported in liquid nitrogen to the laboratory where they were stored at −80 °C until needed in subsequent experiments. Samples were authenticated by Prof. Huasheng Peng (Anhui University of Chinese Medicine).

### Genomic DNA extraction and detection

The genomic DNA was extracted from young leaves of *A. lancea* by means of an improved CTAB method [22]. Approximately 100 mg of fresh leaf samples were frozen in liquid nitrogen and ground into a fine powder with a mortar and pestle. A modified CTAB extraction buffer (2% CTAB, 100 mM Tris-HCl pH = 8.0, 1.4 M NaCl, 20 mM EDTA pH = 8.0, 2% β-mercaptoethanol and 3% polyvinylpyrrolidone) was added and the mixture incubated for 90 min at 65 °C. After incubation, an equal volume of chloroform: isoamyl alcohol (24:1) was added, mixed thoroughly, and centrifuged for 5 min at 12,000 rpm. After centrifugation, the supernatant was transferred to a fresh tube, and isopropanol was added to precipitate the DNA. The extracted DNA was stored in TE buffer (10 mM Tris-HCl, 1 mM EDTA) at −20 °C. DNA concentration was determined using a NanoDrop 2000 spectrophotometer (Thermo Scientific, Wilmington, Delaware, U.S.A.), and purity was verified using the ratios OD_260_/OD_280_ and OD_260_/OD_230_. The integrity and quality of the extracted DNA was verified using 1% agarose gel electrophoresis. For quality control, the DNA samples were randomly selected and sent to independent services for DNA sequencing analysis.

### Library construction and sequencing

The DNA extracted from *A. lancea* was frozen on dry ice and sent to the Beijing Genomics Institute Co., Ltd. (Shanghai, China) for library construction and sequencing. The DNA was sheared randomly into small fragments and sequencing was performed using an Illumina HiSeq 2500 sequencing platform (Illumina, CA, U.S.A.). The clean reads were obtained by clusters passing filter to remove low-quality bases and adapter contamination. The high-quality clean data obtained were used for subsequent analyses.

### 17-mer analysis

Before genome assembly, the genome size, heterozygosity rate, and repeated sequences of *A. lancea* were determined to be consistent with the estimated genome size based on K-mer analysis. K-mer spectrum reflects the heterozygosity of the genome. K-mer analysis of *A. lancea* genome was conducted using *jellyfish* (version 2.1.4). The software *GenomeScope* (http://qb.cshl.edu/genomescope/) was used to fit the 17-mer spectrum and estimate the genome size.

### Heterozygosity prediction and GC content analysis

De novo assembly of the genome was performed using *SOAPdenovo* software (version 2.04). Contigs and scaffolds were constructed to obtain the original genome sequence. The average depth and GC content of each window were calculated to produce the GC-depth plot and repeat content of the genome was determined according to the stratification of GC clusters.

### Preliminary assembly of genome

Preliminary genome assembly and stitching was performed using *SOAPdenovo* software. K-mers were counted for each sequence read, and the frequency of each K-mer was determined. The assembled genome sequences were compared with the original data to determine GC content, contig coverage depth, length, and quantitative distribution of the assembled sequences.

### SSR identification

*MISA* software (version 1.0, http://pgrc.ipk-gatersleben.de/misa/) was used to identify SSR markers. The criteria for identifying SSR motifs were as follows: the minimum number of nucleotide repeats was ten for mononucleotide repeats, six for dinucleotide repeats, and five for trinucleotide, tetranucleotide, pentanucleotide, or hexanucleotide repeat motifs.

### Gene prediction and annotation

Clean reads were processed using *Trinity* software (https://github.com/trinityrnaseq/trinityrnaseq/wiki) to obtain the high-quality unigene library. The obtained unigene was analyzed using bioinformatics, including functional annotation and classification. The BLASTx comparison tool was used to compare the unigene with the protein database (E-value ≤ 1e^−5^). Functional annotation was based on similarity of the gene to the functional annotation information for the unigene encoded protein. The protein databases included NR (non-redundant protein database), COG (Cluster of Orthologous Groups), GO (Gene Ontology database), and KEGG (Tokyo Encyclopedia of Genes and Genomes).

## Results

### Sequencing data statistics

The Illumina HiSeq™ 2500 platform was used for high-throughput, paired-end sequencing to obtain 256.35 Gb of *A. lancea* raw bases. *SOAPnuke* software (version 1.6.5) was used to filter the original sequencing data, and a total of 251.12 Gb clean reads were generated after filtering out low-quality reads, joint contaminations, and PCR duplications. The sequencing depth was 60× coverage (Supplementary Table S1).

### 17-mer analysis and estimation of genome size and heterozygosity

**Figure 1:**
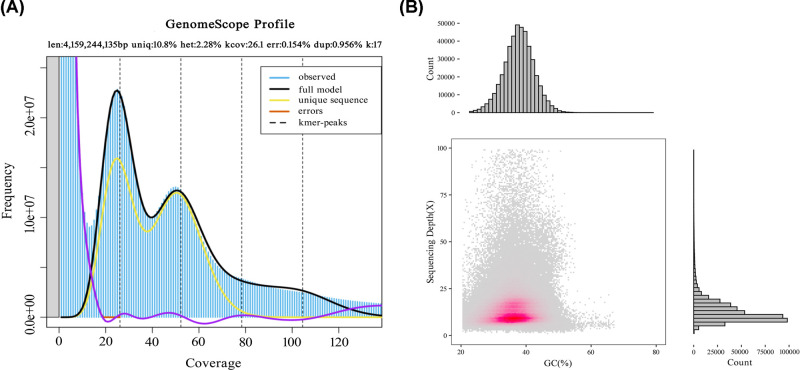
The 17-mer and GC-depth distribution of *A. lancea* genome (**A**) The distribution curve of 17-mer. (**B**) The GC-depth distribution of *A. lancea* genome.

Based on the K-mer analysis, 17-mer was selected for analysis, and the *jellyfish* software was used to quickly count 17-mer frequencies (Table 1). *GenomeScope* software was then used to fit the spectrum of 17-mers, and a total of 251.12 G second-generation data were selected from 16 libraries. The *A. lancea* genome has high heterozygosity and repetitive sequence content consistent with a complex genome (Figure 1A). The 17-mer frequency distribution was ∼26.1, and the total K-mer count was 4,159,244,135 bp. The genome size of non-repetitive sequences was estimated to be 291.49 Mb, which was approximately 60.60% of the *A. lancea* genome. The low K-mer frequency indicated the error rate was 0.154%.

**Table 1:** Statistical data from the 17-mer analysis

	K-mer number	Genome size (Mp)	Repeat (%)	Heterozygous ratio (%)	Used bases (Gp)	Used sequence depth (X)
17	223162157589	4159	89.2	2.28	251.12	60

### GC content and distribution

The assembled contigs were analyzed to obtain GC content and statistical distribution status (Figure 1B). Contigs were mainly distributed in regions where the GC content was between 20 and 50% and concentrated in regions with ∼40% GC content. The average coverage was between 5× and 50×. In this region, two centers of gravity existed in the contig distribution, with an average coverage of approximately 10× and 20×, respectively. Taken together with the K-mer distribution curve, it was speculated that the two centers of gravity corresponded to a heterozygous peak and a homozygous peak, respectively. In the region with the coverage above 50×, there was also a certain amount of contig distribution. It is possible that this was caused by repetitive sequences thus explaining the high heterozygosity rate and repeat sequence ratio of the *A. lancea* genome resulting in difficulties in splicing. According to the GC depth scatter diagram, the GC content of the *A. lancea* genome was estimated to be approximately 38.40%, which was higher than that of *Gastrodia elata* (33.4%), *Scutellaria baicalensis* (34.3%) and *Platycodon grandifloras* (36.3%), and lower than that of *Sorghum bicolor* (42.8%) and *Zea mays* (46.8%), but similar to that of *Rosa roxburghii* (38.4%) and *Helianthus annuus* (38.9%). Therefore, the *A. lancea* genome was of mid-GC content.

### Results of preliminary assembly of genomic data

The 251.12 Gb clean bases were used for preliminary genome assembly after the sequence data were filtered to obtain the preliminary genome sequence. An important indicator of the quality of genome assembly is the Contig N50 value (Table 2). In the *A. lancea* genome, the longest assembled sequence had a length of 289,109 bp, and the length of the N50 was 533 bp. We obtained 4,277,961,587 scaffolds with a total length of 4,508,659,206 bp after further assembly of contigs into scaffolds using *SOAPdenovo* software. The length of the N50 scaffold was 778 bp, and the GC content was 38.4%.

**Table 2:** Genomic information statistics of *A. lancea*

	Scaffold		Contig	
	Length (bp)	Number	Length (bp)	Number
max_len	289109		289109	
N10	4256	68307	2857	94469
N20	2477	211142	1622	299948
N30	1629	438525	1071	629298
N40	1120	775151	750	1110774
N50	778	1260281	533	1790925
N60	526	1967071	375	2751303
N70	335	3047918	253	4135094
N80	190	4851250	165	6261804
N90	119	7855566	112	9405484
Total_length	4508659206	4277961587		
number ≥ 100 bp	12088217	13462724		
number ≥ 2000 bp	311077	199719		
GC_rate	0.367		0.384	

### Genome comparison within the Compositae family

The key word ‘Asteraceae’ (synonymous with Compositae) was used to search the genome database of National Center for Biotechnology Information (https://www.ncbi.nlm.nih.gov/genome/) to obtain the genomic information of 11 Compositae family plants, and this information was compared with the genomic data of *A. lancea* (Supplementary Table S2). The published data show that the genomes of 11 species of Compositae plants ranged in size from 121.712 to 4,159.24 Mb. The genome size of *A. lancea* is 4,159.24 Mb and ranks largest among the Compositae plants, followed by *Helianthus annuus* (3,027.84 Mb), *Chrysanthemum seticuspe* (2,721.84 Mb), and *Lactuca sativa* (2,380 Mb). The Compositae plant with the smallest genome is *Silphium perfoliatum* with a genome size of 3,027.84 Mb (Supplementary Figure S2).

If the GC content is higher than 65% or lower than 25%, it may cause sequence bias on the Illumina sequencing platform, and thus seriously compromise genome assembly [23,24]. The GC content of *A. lancea* genome is 38.4%, which is slightly lower than that of Compositae *Helianthus annuus* and higher than that of other plants in the Compositae family, indicating that the GC content of the *A. lancea* genome is in an acceptable range for genome assembly and thus did not affect the quality of genome assembly. Genome heterozygosity rates were compared across the 12 Compositae plants. The heterozygosity rate of *A. lancea* was the highest (2.28%), followed by *Artemisia annua* (1.0-1.5%) indicating that the genome of *A. lancea* is a complex genome with high heterozygosity and a high repeat ratio.

### SSR markers

**Figure 2:**
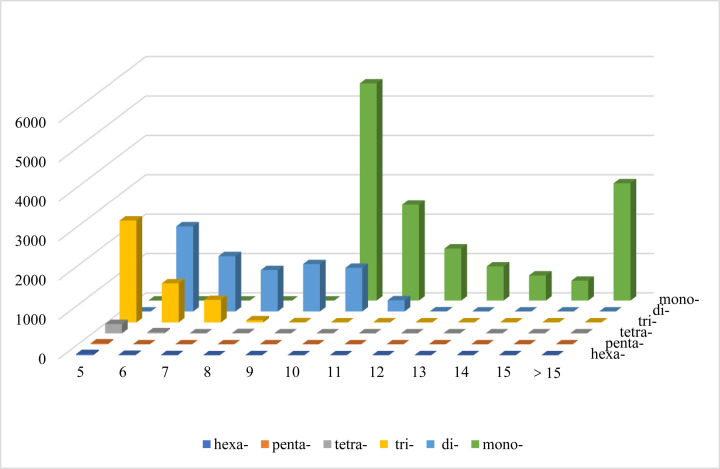
Distribution of various classes of simple repeat motifs with different numbers of repeats in the *A. lancea* genome X-axis, number of SSR repeats; Y-axis, frequency of SSR type.

**Figure 3:**
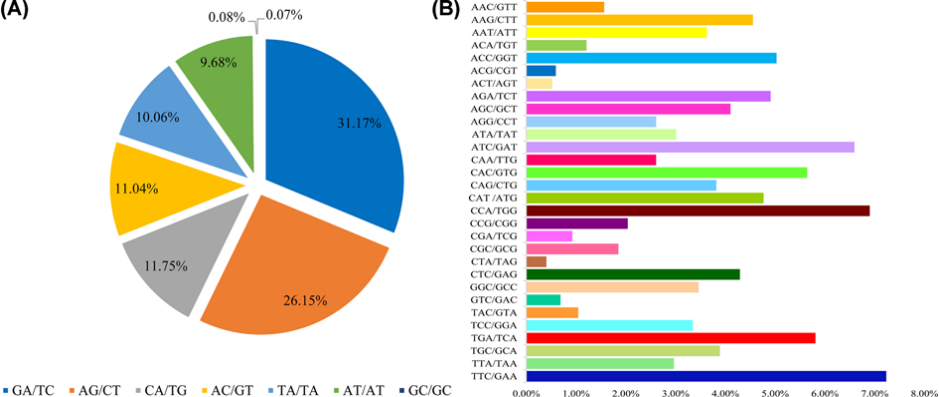
Percentage of different motifs in dinucleotide and trinucleotide repeats in *A. lancea* (**A**) Frequency of different dinucleotide SSR motifs. (**B**) Frequency of different trinucleotide SSR motifs.

A total of 26,034 SSRs were identified, and the most abundant type of repeat was the mononucleotide, which accounted for 54.74% of the observed SSRs, followed by dinucleotide (27.73%), trinucleotide (16.20%), tetranucleotide (1.05%), pentanucleotide (0.10%), and hexanucleotide (0.19%) repeats (Table 3). The most common repeat type was repeated ten times (6620, 25.43%), and the second most common repeated six times (3193, 12.26%) (Figure 2). Among the dinucleotide motifs, GA/TC was the most abundant (31.17%), followed by AG/CT (26.15%), CA/TG (11.75%), AC/GT (11.04%), TA/TA (10.06%), AT/AT (9.68%), GC/GC (0.08%), and CG/CG (0.07%) repeats. The predominant trinucleotide motifs, TTC/GAA, CCA/TGG, and TGA/TCA repeats accounted for 7.23, 6.90, and 5.81%, respectively (Figure 3).

**Table 3:** SSR types detected in the *A. lancea* sequences

Searching item	Number	Ratio (%)
total_SSR_ number	27582	
total_SSR_ length (bp)	476561	
Number of SSR-containing sequences	25144	91.16%
Number of sequences containing more than one SSR	2212	8.02%
Total number of identified SSR	26034	100.00%
Mononucleotide	14250	54.74%
Dinucleotide	7219	27.73%
Trinucleotide	4217	16.20%
Tetranucleotide	273	1.05%
Pentanucleotide	26	0.10%
Hexanucleotide	49	0.19%

### Gene prediction and annotation

A total of 93,434 unigenes (66% of all unigenes) were annotated (Supplementary Table S3), where the TrEMBL database had the greatest match (92,046 unigenes were annotated, 65%), followed by the NR database (91,165, 64%) and Swiss-Prot database (60,411, 42%). A total of 48,493 putative genes were classified into KOG functional categories. The general function prediction only represented the largest group (10,836; 22.35%), followed by signal transduction mechanisms (4,603; 9.50%) and post-translational modification, protein turnover, and chaperones (4,579; 9.44%) (Supplementary Figure S3).

**Figure 4:**
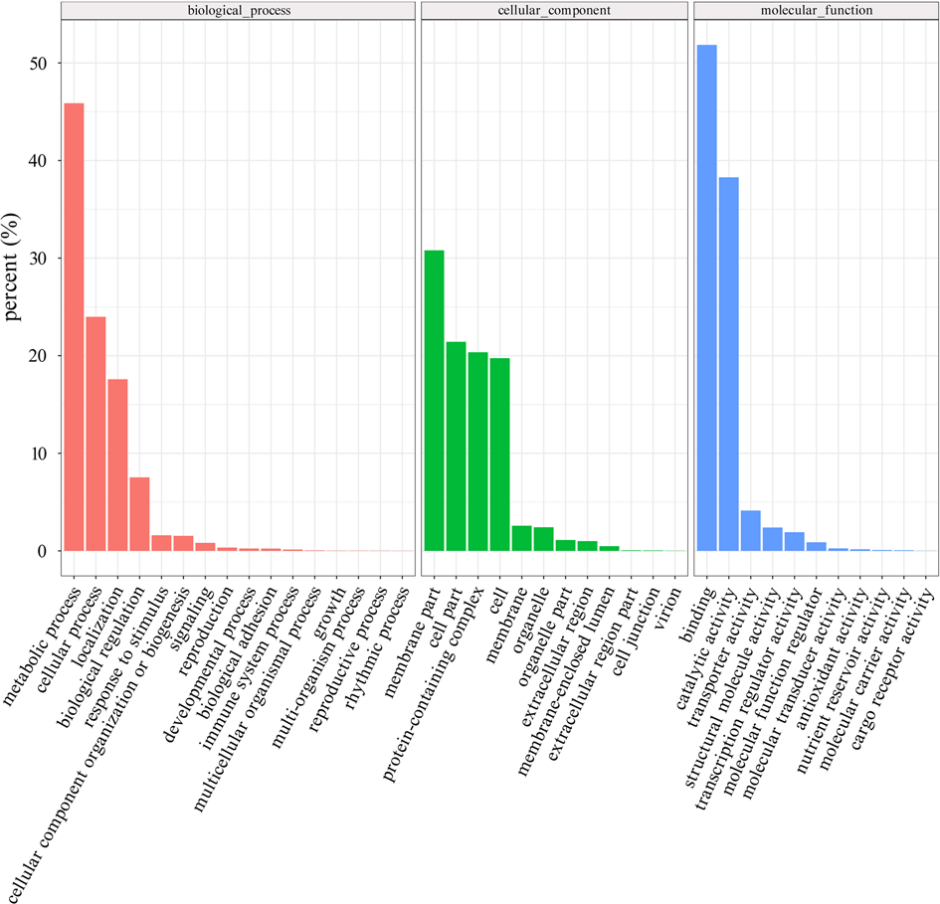
GO functional classification of *A. lancea* unigenes

Based on sequence homology, the 33,896 assembled transcripts were assigned GO terms and further classified into the categories of biological process, cellular component, and molecular function (Figure 4). Among the biological processes, the most represented category was metabolic processes (45.87%), followed by cellular processes (23.98%), and localization processes (17.59%). When sorted based on cellular component, the membrane was the most represented category (30.79%), followed by intracellular (21.42%) and protein-containing complexes (20.35%). For molecular function, the two most represented categories were binding (51.84%) and catalytic activity (38.28%).

**Figure 5:**
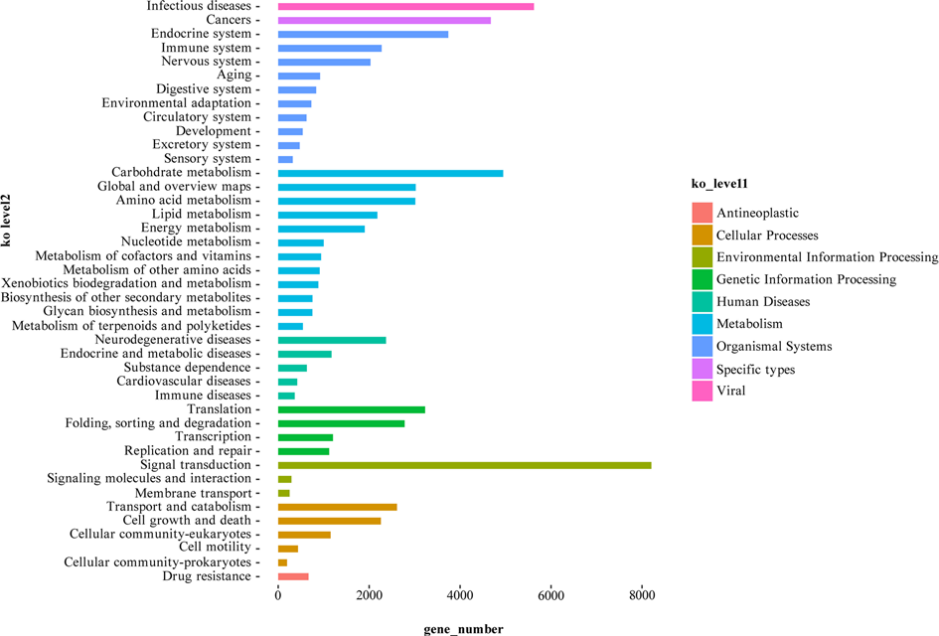
KEGG functional classification of *A. lancea* unigenes

There were 34,929 putative genes assigned to 42 KEGG pathways. A total of 20,862 genes were associated with 12 metabolic pathways; 4,948 (23.72%) were involved in carbohydrate metabolism, followed by global and overview maps (3,025; 14.5%), amino acid metabolism (3,015; 14.5%), and lipid metabolism (2,181; 10.5%). A total of 12,486 genes were associated with organismal systems pathways, 8,749 genes with environmental information processing, 8,336 genes with genetic information processing, 6,654 genes with cellular processes, 5,624 genes with viral diseases and pathways, 4,957 genes with human diseases, and 675 genes with other specific processes and pathways (Figure 5).

The 91,165 unigenes with NR annotation were compared with other species (Supplementary Figure S4). The transcriptome of *Cynara cardunculus* had the largest number of genes similar to the *A. lancea* transcriptome, accounting for 50.78%, followed by the *Lactuca sativa* (9.96%), *Homo sapiens* (9.88%), and *Rhizoctonia solani* (8.39%).

## Discussion

Asteraceae (Compositae) is the largest family of angiosperms in terms of numbers of species, which include many species of significant medicinal and ornamental value. Its special inflorescence composition, corolla type, and other morphological characteristics have important taxonomic significance [25]. *A. lancea* can be divided into two types, namely the Maoshan and Dabieshan types [26,27]. The Maoshan type is distributed primarily in the Maoshan area of Jiangsu Province. However, in recent years, the wild *A. lancea* in Jiangsu province has been gradually becoming endangered and has been listed as one of the four endangered medicinal plants in Jiangsu province [28]. Therefore, the study of the *A. lancea* genome will have important ecological significance.

In the current study, the whole genome of *A. lancea* was sequenced for the first time. Sequencing and analysis of the genome was performed using the NGS method, and the genome size was automatically estimated to be 4.16 Gb using *GenomScope* software. The GC content of the *A. lancea* genome was 38.4%, and the heterozygosity rate and repeat sequence ratios were 2.28 and 89.2%, respectively. The high heterozygosity rate and repeat sequence content indicates a complex genome. To obtain a high-quality whole genome map of *A. lancea*, a strategy combining the PacBio and Illumina sequencing platforms, supplemented by the High-throughput Chromosome Conformation Capture (Hi-C) technique is recommended. Genome size, also known as C-value or Constant-value, is an important genetic feature of an organism [29]. The genome 2C-value of *A. lancea* was estimated previously by Deng et al. to be 9.73 pg (1 pg = 978 Mbp) by flow cytometry [8], which was close to the results of our genome survey. There are great differences in genome size among species within the Compositae family. Vinogradov [30] showed that the genomes of threatened plants (whose populations are now on the decline) may actually be larger than expected compared with those of their relatives. The genome of *A. lancea* ranks first in the Compositae family in regard to size at 4,159.24 Mb. This shows that *A. lancea* is more endangered than other plants of the Compositae family.

The genomic SSR analysis of *A. lancea* showed that there were great differences in the SSR content. The SSR types of *A. lancea* were abundant and with high frequencies of mononucleotide, dinucleotide, and trinucleotide repeats. This is similar to plants such as *Dioscorea zingiberensis* [31], *Rosa roxburghii* [32], *Dracaena cambodiana* [33], and *Apocynum venetum* [34], but not consistent with the majority of plants where dinucleotide and trinucleotide repeats are the main types of SSR [35–37]. Among the dinucleotide repeat motifs, the GA/TC and AG/CT repeats were the two most abundant types, which accounted for 31.17 and 26.15%, respectively, followed by CA/TG (11.75%). Among the trinucleotide repeat motifs, the TTC/GAA was most abundant, which accounted for 7.23%, followed by CCA/TGG (6.90%) and TGA/TCA (5.81%). The SSRs of *A. lancea* are polymorphic, thus laying a foundation for using these SSR molecular markers in screening for genetic diversity.

A total of 93,434 *A. lancea* genes, 66% of the *A. lancea* genome, matched known genes in common databases. It is speculated that the reason for the remaining uncommented genes may be due to either non-coding or incomplete sequences [38]. According to the NR annotation, *Cynara cardunculus* genes were the most commonly annotated with *A. lancea* indicating that *A. lancea* has the closest relationship with *Cynara cardunculus*. Comparing the functional analyses of the *A. lancea* genome using the KOG, KEGG, and GO classification systems, it was found that the number of transcripts annotated to KOG was the largest, with the most abundant genes related to general function, signal transduction mechanism and post-translational modification, protein turnover, and chaperones. The results of the present study will play an important role in future whole-genome sequencing projects and provide a rich resource for future functional studies, which will greatly enhance our understanding of the genetic regulatory mechanisms of *A. lancea*.

## Supplementary Material

Supplementary Figure S1-S4 and Tables S1-S3

## Abbreviations

GO: Gene Ontology database
KEGG: Kyoto Encyclopedia of Genes and Genomes
KOG: euKaryotic Clusters of Orthologous Groups
NGS: next-generation sequencing
NR: non-redundant protein database
SSR: simple sequence repeat
