# Supplementary material for: Genome survey sequencing of Atractylodes lancea and identification of its SSR markers
Source: Biosci Rep. 2020 Oct 28;40(10):BSR20202709. doi: 10.1042/BSR20202709 (PMC7593537; doi:10.1042/BSR20202709)
Supplement: Supplementary Figure S1-S4 and Tables S1-S3 [file BSR-2020-2709_supp.pdf]

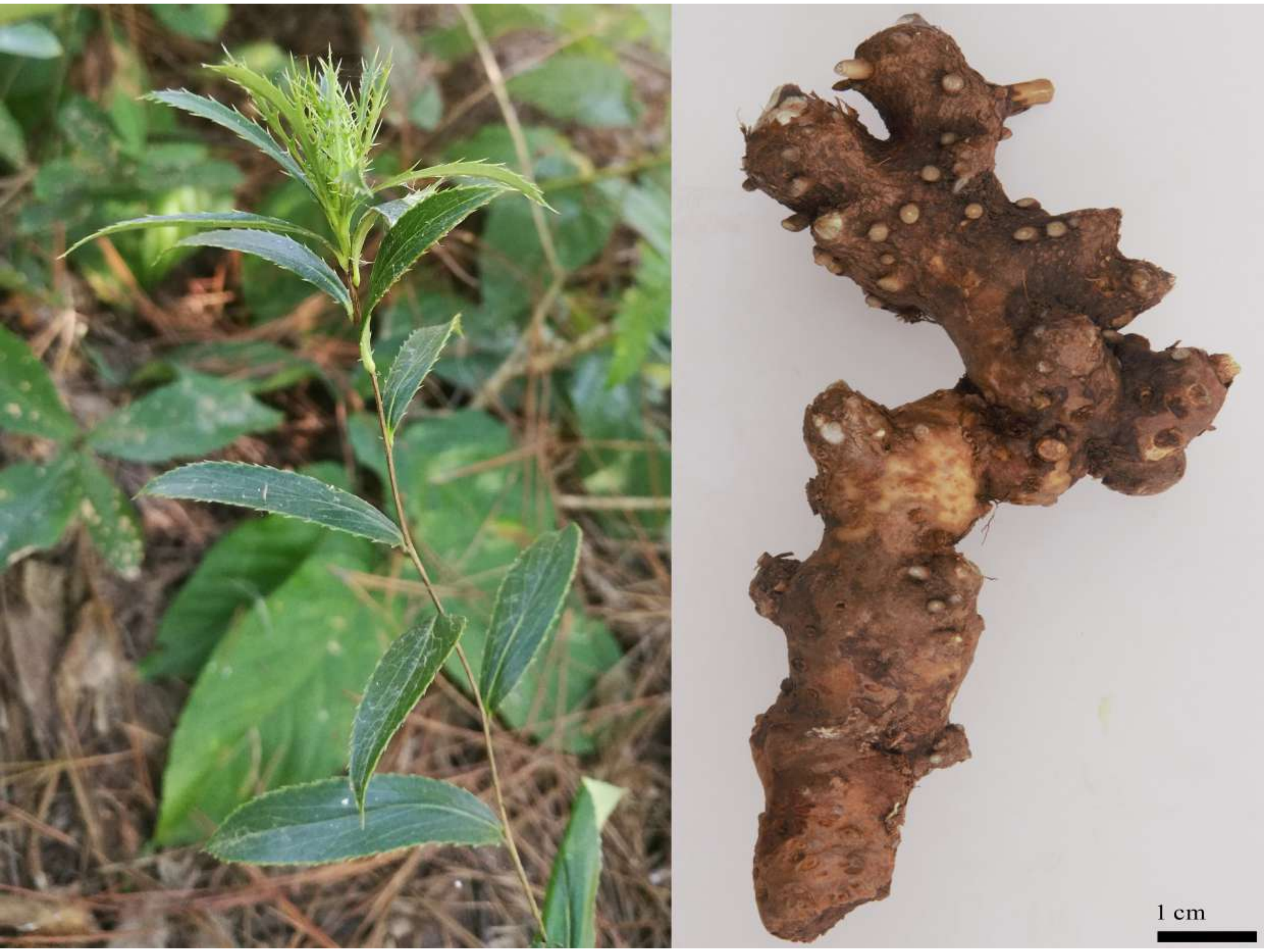
1 cm

Supplementary Figure S1. Plants and rhizome of *A.lancea*

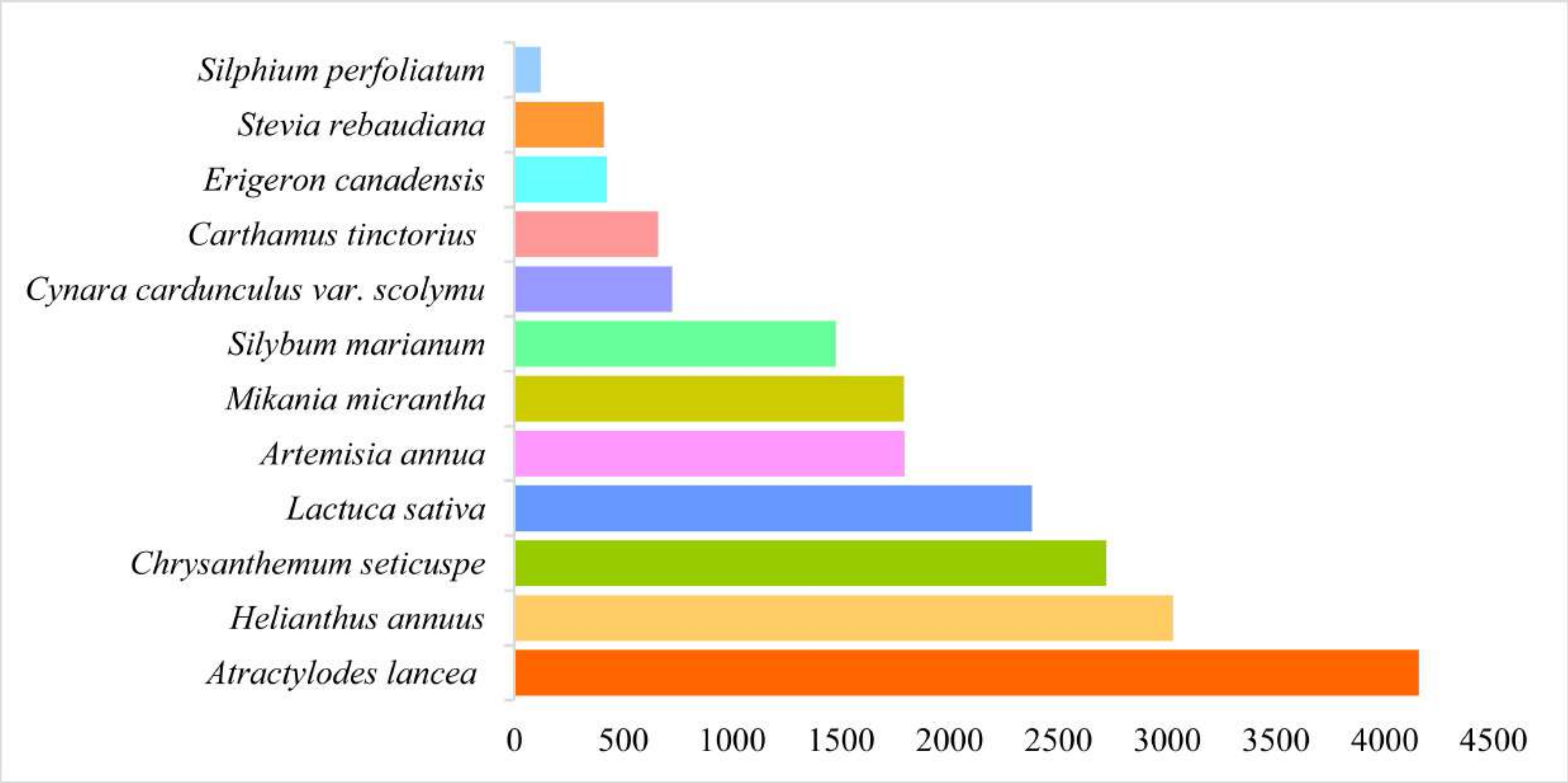
Silphium perfoliatum
Stevia rebaudiana
Erigeron canadensis
Carthamus tinctorius
Cynara cardunculus var. scolymu
Silybum marianum
Mikania micrantha
Artemisia annua
Lactuca sativa
Chrysanthemum seticuspe
Helianthus annuus
Atractylodes lancea
0
500
1000
1500
2000
2500
3000
3500
4000
4500

Supplementary Figure S2. Comparison the genome size of 12 species of Compositae

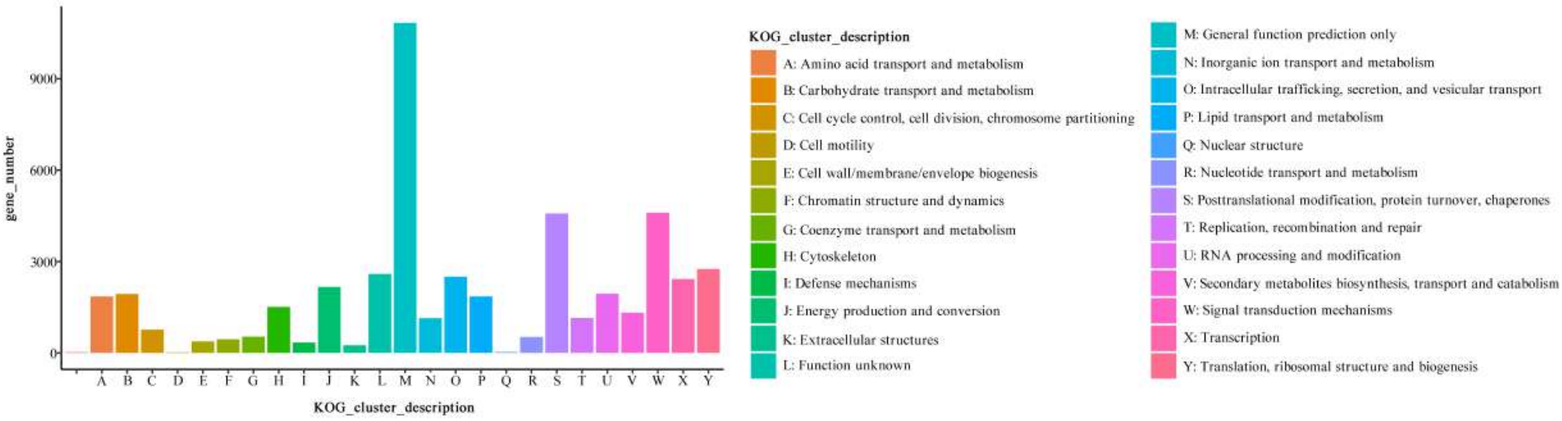
gene_number
9000
6000
3000
0
A B C D E F G H I J K L M N O P Q R S T U V W X Y
KOG_cluster_description
KOG_cluster_description
A: Amino acid transport and metabolism
B: Carbohydrate transport and metabolism
C: Cell cycle control, cell division, chromosome partitioning
D: Cell motility
E: Cell wall/membrane/envelope biogenesis
F: Chromatin structure and dynamics
G: Coenzyme transport and metabolism
H: Cytoskeleton
I: Defense mechanisms
J: Energy production and conversion
K: Extracellular structures
L: Function unknown
M: General function prediction only
N: Inorganic ion transport and metabolism
O: Intracellular trafficking, secretion, and vesicular transport
P: Lipid transport and metabolism
Q: Nuclear structure
R: Nucleotide transport and metabolism
S: Posttranslational modification, protein turnover, chaperones
T: Replication, recombination and repair
U: RNA processing and modification
V: Secondary metabolites biosynthesis, transport and catabolism
W: Signal transduction mechanisms
X: Transcription
Y: Translation, ribosomal structure and biogenesis

Supplementary Figure S3. KOG functional classification of *A．lancea* unigenes.

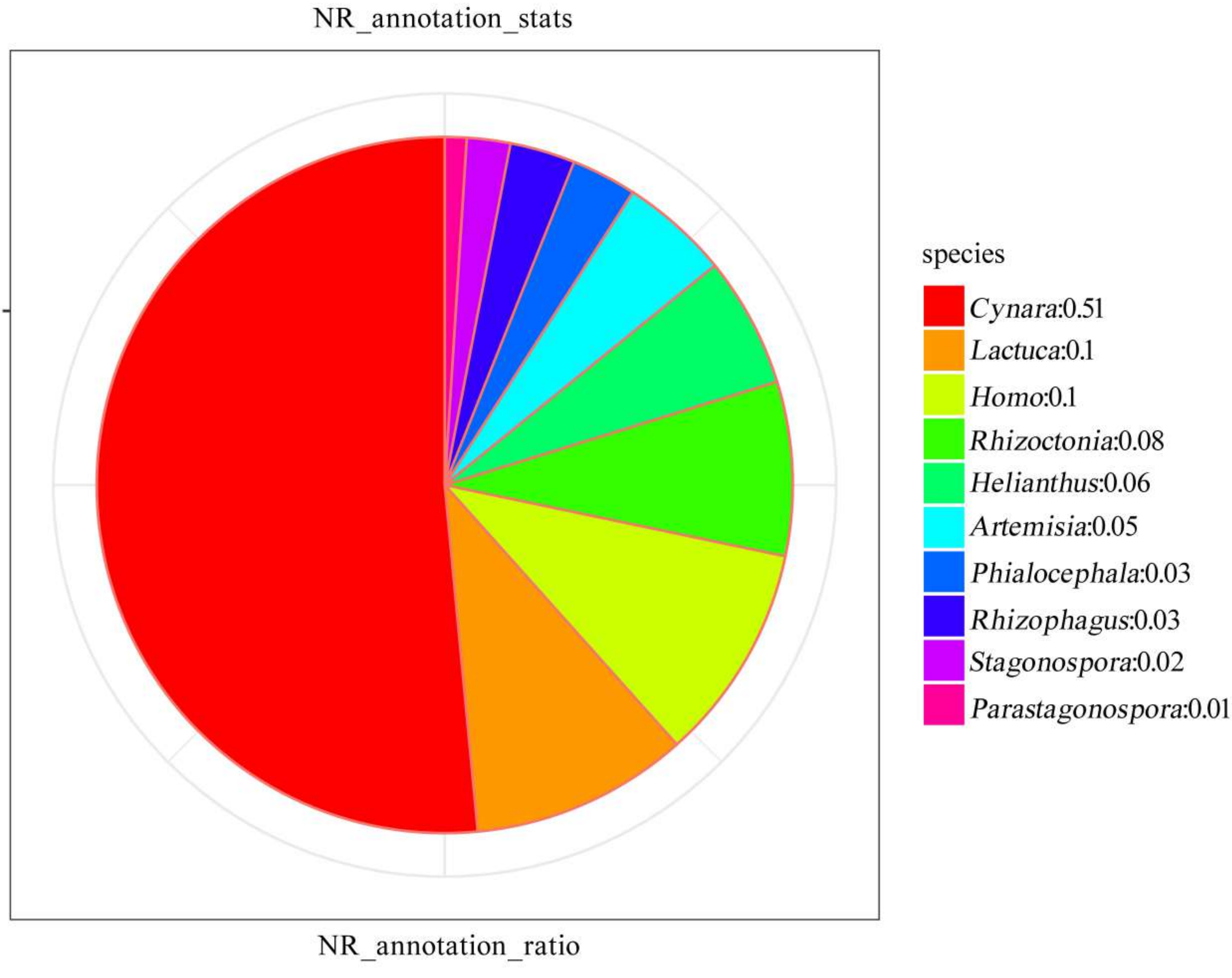
NR_annotation_stats
NR_annotation_ratio
species
Cynara:0.51
Lactuca:0.1
Homo:0.1
Rhizoctonia:0.08
Helianthus:0.06
Artemisia:0.05
Phialocephala:0.03
Rhizophagus:0.03
Stagonospora:0.02
Parastagonospora:0.01

Supplementary Figure S4. NR annotated homologous species distributions

Supplementary Table S1. Statistics of filtering data

Lib id	Length	Raw data	Clean data	GC rate
190626_I9_V300022484_L3_RHIs hvDAAAA-573	150	14,518,852,200	14,249,431,800	38.98
190626_I9_V300022484_L3_RHIs hvDAAAA-574	150	16,785,220,500	16,471,137,000	38.91
190626_I9_V300022484_L3_RHIs hvDAAAA-575	150	14,729,106,000	14,448,511,200	38.9
190626_I9_V300022484_L3_RHIs hvDAAAA-576	150	13,615,597,500	13,348,510,200	38.93
190626_I9_V300022484_L3_RHIs hvDAAAA-577	150	15,861,435,600	15,566,351,400	38.89
190626_I9_V300022484_L3_RHIs hvDAAAA-578	150	16,151,568,000	15,851,755,800	38.94
190626_I9_V300022484_L3_RHIs hvDAAAA-579	150	17,966,042,100	17,646,709,500	38.87
190626_I9_V300022484_L3_RHIs hvDAAAA-580	150	14,990,523,000	14,716,546,800	38.94
190626_I9_V300022484_L4_RHIs hvDAAAA-573	150	15,333,206,400	14,993,685,600	38.88
190626_I9_V300022484_L4_RHIs hvDAAAA-574	150	17,745,398,100	17,352,470,700	38.8
190626_I9_V300022484_L4_RHIs hvDAAAA-575	150	15,459,255,300	15,108,670,500	38.81
190626_I9_V300022484_L4_RHIs hvDAAAA-576	150	14,491,109,700	14,161,733,400	38.8
190626_I9_V300022484_L4_RHIs hvDAAAA-577	150	16,822,270,200	16,451,535,000	38.78
190626_I9_V300022484_L4_RHIs hvDAAAA-578	150	17,067,861,600	16,689,940,500	38.84
190626_I9_V300022484_L4_RHIs hvDAAAA-579	150	18,935,608,800	18,532,550,100	38.77
190626_I9_V300022484_L4_RHIs hvDAAAA-580	150	15,874,163,700	15,529,107,600	38.84

Supplementary Table S2. Genome assembly statistics of *A. lancea* and comparisons to 11 genomes of Compositae

Species	Gene size (Mb)	Number of protein-coding genes	GC content (%)	Hetero zygosity (%)	Repeat (%)	References
Atractylodes lancea	4159.24	n.c	38.4	2.28	89.2	This study
Carthamus tinctorius (safflower)	661.938	n.c	37.3	n.c	n.c	Bower, J.E, 2016
Stevia rebaudiana	411.38	n.c	34.6	n.c	n.c	n.c
Chrysanthemum seticuspe	2721.84	n.c	36.1	n.c	72.5	Hideki, 2019
Silphium perfoliatum	121.712	n.c	36.7	n.c	n.c	n.c
Silybum marianum	1477.58	n.c	37.2	n.c	n.c	n.c
Erigeron canadensis	425.611	n.c	34	0.203	42.9	Laforest et al., 2020 Peng et al., 2014
Mikania micrantha	1790.64	46329	36.2	n.c	n.c	n.c
Cynara cardunculus var. *scolymus*	725.198	38406	36.75	n.c	n.c	Caglione et al., 2016
Lactuca sativa	2380	38919	37.75	n.c	n.c	Verwaaijen et al., 2017 Reye et al., 2016
Artemisia annua	1792.86	66918	34.1	1.0-1.5	60.1	Shen et al., 2018
Helianthus annuus	3027.84	73839	38.91	n.c	n.c	Bock et al., 2014 Badouin et al., 2017

Supplementary Table S3. Statistics of gene functional annotation

Data_base	annotated_number	annotated_ratio
GO	33896	24%
KEGG	34929	24%
KOG	48493	34%
nr	91165	64%
pfam	58121	41%
swiss_prot	60411	42%
TrEMBL	92046	65%
Total	93434	66%
